# Protective Effect of Potential Probiotic Strains from Fermented Ethiopian Food against *Salmonella* Typhimurium DT104 in Mice

**DOI:** 10.1155/2020/7523629

**Published:** 2020-04-13

**Authors:** Guesh Mulaw, Diriba Muleta, Anteneh Tesfaye, Tesfaye Sisay

**Affiliations:** ^1^Department of Microbial, Cellular and Molecular Biology, College of Natural and Computational Sciences, Addis Ababa University, Addis Ababa 1176, Ethiopia; ^2^Biology Department, College of Natural and Computational Sciences, Aksum University, Axum 1010, Ethiopia; ^3^Institute of Biotechnology, Addis Ababa University, Addis Ababa 1176, Ethiopia

## Abstract

*Salmonella* is one of the most harmful pathogens responsible for foodborne outbreaks, illnesses and deaths. The aim of this study was to evaluate the effect of potentially probiotic strains against *Salmonella* Typhimurium DT104 in mice. The compatibility test among the selected potential probiotic strains (*Lactobacillus plantarum* K132*, Lactobacillus paracasei* K114 and *Lactococcus lactis* E124) using the cross-streaking method showed the absence of antagonism. The anti-*Salmonella* activities of coculture of the isolated potential probiotics in the form of mixed or single culture showed a remarkable anti-*Salmonella* activity with 96.50 to 100% growth inhibition. The combination of strains, which showed the highest growth inhibition rates against *Salmonella* Typhimurium DT104, was used to test their effect on the colonization of mice by *Salmonella* Typhimurium DT104. White albino male mice were pretreated with the mixed potential probiotics for 7 days and infected with *Salmonella* Typhimurium DT104 for 1 day. A total of 3 treatments were applied, during which the negative control group was treated with phosphate-buffered saline (PBS); a positive control group (typ) was challenged with *Salmonella* Typhimurium DT104 alone. The treated group (pro-typ) was pretreated with mixed potential probiotic culture and then infected with *Salmonella* Typhimurium DT104. The survival rate of mice and counts of *Salmonella* in feces were recorded. The survival rate of mice on day 21 after the oral challenge with *Salmonella* Typhimurium DT104 was significantly (*p* < 0.05) higher in the experimental pro-typ group (100% survival) compared with the positive control group (20% survival). The counts (colony-forming unit per ml) of *Salmonella* in feces were significantly lower (*p* < 0.05) for the pro-typ group compared to the typ group. The combination of potential probiotic strains was able to protect mice against *Salmonella* Typhimurium DT104 infection that demonstrates their potential to be used as probiotic cultures for the production of functional fermented products.

## 1. Introduction

Foodborne diseases (FBDs) pose a severe public health problem that significantly affects people's wellbeing and leads to serious socioeconomic implications [1]. The major foodborne bacterial pathogens are *Campylobacter jejuni*, *Clostridium perfringens*, *Escherichia coli*, *Listeria monocytogenes, Salmonella* spp. and *Staphylococcus aureus* [2]. These pathogens have developed multiple drug resistance and cause great economic losses in developing as well as developed countries [3]. The problem of foodborne diseases is multifactorial, and their prevention and control require multidisciplinary approaches [4].

Among the major foodborne pathogens, *Salmonella enterica* is one of the leading causes of serious illness ranging from acute gastroenteritis to systemic infections including typhoid [5]. Oral infection with *Salmonella* Typhimurium in mice provokes a disease similar to that caused by *Salmonella* Typhi in humans, with fever, enteritis, and septicemia which is lethal to the host [6]. However, the nature and severity of the infection developed depends on many factors, including the serovar involved, the virulence of the strain, the infective dose, the age and immune status of the host. Therefore, it is estimated that *Salmonella* species cause 93.8 million gastroenteritis infections worldwide and resulted in 155 000 deaths each year [7].

Currently, vaccination and antibiotics are used to prevent and control *Salmonella* infections. Accordingly, antibiotic applications are the common clinical treatments for *Salmonella* infection which, in turn, promotes the development of resistant *Salmonella* species towards antibiotics [8]. In addition, the prolonged use of antibiotics leads to changes in the intestinal commensal microflora [9]. Due to the occurrence of multidrug-resistant strains and the suboptimal efficacy of currently available vaccines, alternative intervention strategies against *Salmonella* infections are urgently needed [10–12]. One of the promising alternative control approaches is the possible beneficial use of probiotics against various pathogens, including *Salmonella* spp. [13].

The consumption of a large number of probiotics (live microorganisms) together with food item can fundamentally promotes the health of the consumers [14]. The possible mechanisms, by which probiotics protect against enteric pathogen infections, are the production of antimicrobial substances, competition for limited resources, and antiadhesive effects [15]. A great number of *in vivo* and *in vitro* studies have been carried out to evaluate the effect of probiotics in the prevention and treatment of gastrointestinal infections caused by *Salmonella* species [16–18]. The beneficial effects of the probiotics are known to be genus, species and strain specific [19–21]. Currently, food-based probiotics have supposed greater significance as different food products can harbor native and beneficial probiotics and therefore can be used for both nutritional and therapeutic purposes [22].

Ethiopian fermented food products are well known for their unique fermentation style and can be used as a source of potentially beneficial probiotics. Tesfaye et al. [18] revealed the antagonistic effect of lactic acid bacterial strains either as pure or defined mixed cultures against some foodborne pathogens during fermentation and storage of fermented milk. There are still few research data available on the characterization of probiotic LAB from Ethiopian traditional fermented foods. Most of the traditionally fermented products of Ethiopia are consumed without further heat processing which can be considered as ideal vehicles to carry probiotic bacteria into the human gastrointestinal tract. Probiotic strains isolated from traditionally fermented foods and drinks could have a desirable functional property for their application as probiotics against foodborne pathogens. Thus, the main objective of this study is to test the effect of three potentially probiotic strains of LAB isolated from traditionally Ethiopian fermented *ergo* and *kocho* products against *Salmonella* Typhimurium under *in vivo* conditions using laboratory animal.

## 2. Materials and Methods

### 2.1. Bacterial Strains and Growth Condition

The bacterial strains and sources of isolation used in this study are listed in Table 1. The potential probiotic strains were isolated from traditionally fermented *kocho* and *ergo* products. These strains were identified as *Lactobacillus plantarum* K132*, Lactobacillus paracasei* K114 and *Lactococcus lactis* E124 by whole genome sequencing in the Earlham Institute (Norwich, UK) (unpublished data). All of the strains were Gram-positive and catalase-negative and were able to grow on MRS agar. *Salmonella* Typhimurium DT104 was obtained from Ethiopian Public Health Institute (EPHI), Addis Ababa, Ethiopia (Table 1). *Salmonella* Typhimurium DT104 was able to grow aerobically in xylose-lysine-deoxycholate (XLD) agar for 24 h at 37^o^C.

### 2.2. Compatibility between Probiotic Strains

The compatibility of the selected potential probiotic strains was examined by a cross-streak method as previously described by Pedersen and Tannock [23]. Overnight cultures of the isolates were streaked perpendicularly by forming across with each other on MRS agar plates. The plates were incubated at 37^o^C for 48 hours anaerobically using an anaerobic jar. The type of growth in the confluence zones (stimulation, inhibition, or absence of interaction between the strains) was visually determined [24]. The presence of growth inhibitory halos indicates incompatibility between strains.

### 2.3. Coculture Assay

To evaluate the effect of selected potential pure strains (*Lactococcus lactis* E124*, Lactobacillus paracasei* K114 and *Lactobacillus plantarum* K132) and their combinations on the growth of *Salmonella* Typhimurium DT104, liquid coculture technique was used as described by Potočnjak et al. [25] with some modifications. Before cocultivation, the selected strains and *Salmonella* Typhimurium DT104 were grown separately in de Man, Rogosa, and Sharpe (MRS) broth and tryptic soy broth (TSB), respectively. Thus, 100 *μ*l of each and mixed strains (a total of 10^6^ CFU/mL) and of S. Typhimurium (10^4^ CFU/mL) were inoculated into brain heart infusion (BHI) broth and incubated for 24 h at 37°C in aerobic conditions. The control was a monoculture of *S*. Typhimurium. The number of *Salmonella* was determined by plate count agar on the xylose-lysine-deoxycholate (XLD) solid medium. Experiments were carried out in triplicate. The inhibition percentage was calculated according to the following equation [26]:

(1) $$\begin{array}{c} \text{inhibition}\left(\%\right)=\frac{\text{CFU }{\text{ml}}^{-1}\text{in control}-\text{CFU }{\text{ml}}^{-1}\text{in co incubation culture}}{\text{CFU }{\text{ml}}^{-1}\text{in control}}\times 100. \end{array}$$

Therefore, the preparation of pure and mixed probiotic LAB cultures for the coculture assay is presented in Table 2.

### 2.4. *In Vivo* Antagonistic Effect of Mixed Probiotic Strains against *Salmonella* Infection

#### 2.4.1. Experimental Mice

Four to six weeks aged male white albino mice were obtained from the Animal House of the Department of Microbial, Cellular and Molecular Biology, Addis Ababa University. Mice were housed in cages in the animal room. These mice were provided with standard diet and water *ad libitum*. The bedding of mice was changed every three days, and the health status of the animals was monitored regularly.

#### 2.4.2. Experimental Design

A total of 30 male white albino mice were used in the study. Five mice per cage were randomly congregated into three groups. Group I served as a negative control and was treated with phosphate-buffered saline (PBS). Group II was challenged with mono *Salmonella* Typhimurium DT104 culture (positive control, typ). Protection against *Salmonella* Typhimurium DT104 infection by administration of mixed probiotic cultures was evaluated in Group III (pro-typ). Fecal samples from each group were pooled and checked for a week for the absence/presence of *Salmonella.* Five gram (g) of fecal material was homogenized into 45 ml of sterile 0.1% buffered peptone water and 25 ml was further enriched in 225 ml of tryptic soya broth. Enriched cultures were streaked on XLD plates. After ensuring the absence of *Salmonella* spp. from all groups of mice, *Salmonella* Typhimurium DT104 and various LAB cultures were administered to mice after depriving the mice water a day before as indicated by Truusalu et al. [27].

#### 2.4.3. Treatments Preparation

The best performing potential probiotic strains in a liquid coculture assay were selected for an *in vivo* test using a mouse model. Consequently, *in vivo* evaluation of the probiotic effect of the mixed potential probiotic strains (*Lactococcus lactis* E124*, Lactobacillus paracasei* K114 and *Lactobacillus plantarum* K132) against *S.* Typhimurium DT104 was done via oral inoculation of mice following the protocol given in Chen et al. [28]. The experiment was conducted twice, and the average was used for analysis.

Probiotic cultures were separately grown overnight at 37°C in 10 ml of MRS broth. To prepare the mixed culture, the overnight growth of each culture (10 ml) was quantified by serial dilution and plate counting to get log 6 CFUml^−1^ that achieved by mixing equal volumes of each strain and was divided into daily portions of combined strains. Prior to the feeding step, the mixed culture was prepared by mixing log 6 CFUml^−1^ of each of the potential probiotic strains. Hence, the mixed potential probiotic strains (*Lactobacillus plantarum* K132*, Lactobacillus paracasei* K114 and *Lactococcus lactis* E124) were termed as a multi-strain formula (MFA). *Salmonella* Typhimurium DT104 was grown separately overnight at 37°C in 10 ml of tryptic soya broth. Overnight growth of LAB cultures and cultures of *S.* Typhimurium DT104 were separately serially diluted in 10 ml of sterile 0.1% buffered peptone water to give 10^4^ CFUml^−1^.

The experiment was divided into 3 stages [29]: the initial stage (day 1 to 7), the infection stage (day 8), and the final stage (day 9 to 21). Group I mice were given 0.3 ml of phosphate-buffered saline (PBS) for the whole 7 days. Group II mice were challenged with mono *Salmonella* Typhimurium DT104 at day 8 with a dose (0.3 ml; 4 log CFU·mL^−1^ of viable organism) as one oral dose. Protection against *Salmonella* Typhimurium DT104 infection by administration of mixed probiotic strains was evaluated in Group III. In this Group, mixed LAB strains (0.3 ml per day; 6 log CFU·mL^−1^) were given using a sterile syringe blunt-ended tube for consecutive 7 days and at the day 8, they were orally administered with *Salmonella* Typhimurium DT104 (0.3 ml; 4 logs CFU·mL^−1^ of viable organism) as one oral dose. The symptoms and deaths of mice were registered, and all the survived animals were killed by cervical dislocation on the 21^st^ day. The percent survival (the number of the alive/total number of mice) was recorded every day for 21 days.

#### 2.4.4. Viable Cell Counts of *Salmonella* Typhimurium DT104 in Fecal Material of Mice

Aseptically, freshly voided fecal material of mice was collected daily using sterile forceps from day 9 to day 21. Fecal material (5 g) from each group was moistened for 10 minutes in 45 ml of 0.1% buffered peptone water and then homogenized using a Stomacher lab blender (Stomacher 400, Seward, London, UK). Then, appropriate dilutions of each homogenate (0.1 ml) were plated on xylose-lysine-deoxycholate (XLD) agar for enumeration of *Salmonella* Typhimurium DT104. Plates were incubated at 37°C for 24 hours, and colony-forming units on the plates were recorded. When counts were <log 1 CFU·mL^−1^, samples were enriched in tryptic soya broth. Each determination was done in triplicate.

## 3. Data Analysis

All experiments were carried out in triplicate. The results were expressed as mean standard deviation (SD). Statistical analysis was performed using SAS software R 9.1 (SAS Institute Japan, Tokyo) and Stat View Ver. 5 (SAS Institute, Cary, NC).

## 4. Results

### 4.1. Compatibility among Probiotic Strains

Compatibility among 3 selected potential probiotic strains has been determined by cross-streaking the strains *(Lactobacillus plantarum* K132*, Lactobacillus paracasei* K114 and *Lactococcus lactis* E124) on an MRS agar plate (Table 3). Although the selected probiotic strains were confirmed to have antagonistic activity towards the tested pathogen, the 3 selected probiotics LAB strains did not show any inhibition halos against each other, suggesting the absence of antagonism among strains when combined in mixed cultures (Table 3). Generally, the cross-streak assay showed similar results, as no evidence of competition was noticeable at sites of cogrowth in the solid medium in any combination of probiotic strains assayed (Table 3). Finally, after the compatibility experiment, the 3 potential probiotic strains isolated from traditional fermented food products were taken to the next step for the coculture assay study.

### 4.2. Coculture Assay

All the pure and mixed probiotic LAB strains when separately and/or in combined forms cocultured against the test foodborne pathogen (*Salmonella* Typhimurium DT104) showed more than 96% growth inhibition of the test organism (Table 4). The highest coculture antagonistic activity (100% growth inhibition) was observed with the mixed cultures of the three probiotic strains (mix 4) followed by the combination of two combined probiotic strains in the form of mix 2, mix 3, and mix 1 with 99.74%, 99.72%, and 99.71% of inhibition, respectively. However, the lowest (*p* < 0.05) growth inhibition (96.50%) was shown with the separate pure culture of *Lactobacillus plantarum* against *Salmonella* Typhimurium DT104 (Table 4).

### 4.3. *In Vivo* Antagonistic Effect of Mixed Probiotic Strains Against *Salmonella* Infection

**Figure 1:**
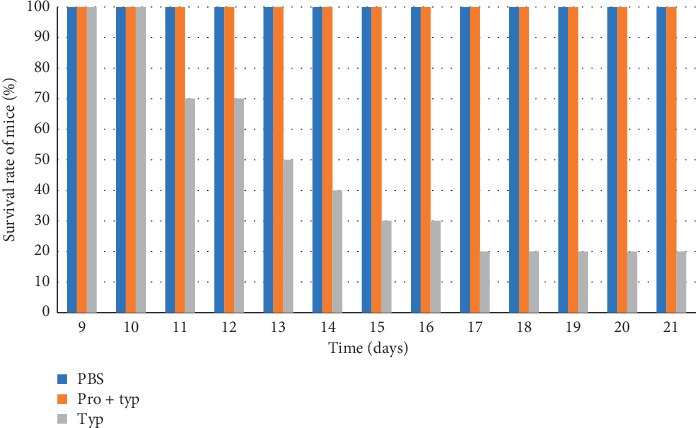
Survival of mice treated or not treated with mixed potential probiotic strains and orally infected with *Salmonella* typhimurium DT104. Means not connected by the same letter are significantly different at *p* < 0.05. Results expressed as average (*n* = 2) ± SD (standard deviation). Legend: PBS = phosphate-buffered saline; pro + typ = both mixed probiotic cultures and *Salmonella*; typ = mono *Salmonella* Typhimurium DT104.

The survival rate of the treated group (pro-typ) was 100%, whereas only 20% of the positive control group that was challenged only with *S.* Typhimurium DT104 survived (Figure 1).

Apparently, day 1 after infection with *Salmonella* Typhimurium DT104, all the mice in the pro-typ group and positive control group (typ) started to show disease symptoms. Ultimately, hair erection and diarrhea were observed. However, the mice pretreated with the mixed potential probiotic strains were able to recover from sickness, but the mice challenged with *Salmonella* Typhimurium DT104 alone (positive control group) became sick and finally died. Therefore, in the positive control group (Group II), the first mortality rate (30%) was observed at day 3 and at day 5 and the death rate was increased to 50%. As testing time extended beyond five days, the mortality rate was increasing in the positive control. Finally, the mortality rate (80%) in mice was recorded at the end of day 21. However, there was no mortality in the negative control Group I only treated with the PBS (Figure 1).

### 4.4. Viable Cell Count of *Salmonella* Typhimurium DT104 in Fecal Material of Mice

In the negative control group (PBS) of mice, no viable *Salmonella* Typhimurium DT104 counts were detected (Table 5). In comparison with the results from Group II, *Salmonella*-challenged mice that fed on mixed probiotic strains for 7 days reduced the *Salmonella* cells in the feces when measured from day 9 to day 21 (*p* < 0.05; Table 5). The findings showed that the CFU count of *Salmonella* Typhimurium DT104 in mice which were given with mixed probiotics strains were lower than that of the positive control group (Table 5). This shows that the presence of combined probiotic LAB in the gut is able to inhibit the growth of the pathogenic bacterium. On the other hand, in the feces of mice fed with combined probiotic LAB strains, the fecal CFU count of *Salmonella* Typhimurium DT104 cells was significantly reduced from 2.30 to 0.00 log CFU·ml^−1^. However, the noticeable effect of treatment with the combined potential probiotic LAB strains was observed from day 18 and onwards (Table 5).

Generally, the counts of *Salmonella* Typhimurium DT104 from the feces of the positive control group (typ) were consistently higher throughout the experiment of postinfection than from the feces of the probiotic-treated mice group (Table 5). Hence, when the selected potential probiotic LAB strains were administered at a dose of log 6 CFUml^−1^ level, *Salmonella* Typhimurium DT104 was eliminated from the feces of the probiotic-treated mice group at day 20 and onwards. Thus, in comparison with the results from Group I, there was no difference observed from days 20 and 21 with the mice treated with mixed probiotic strains since there was no viable cell count of the test pathogen in either (Table 5). But the recovery number of viable cell counts in feces of mice challenged only with *Salmonella* at day 20 was observed to be 4.53 log CFU·ml^−1^.

## 5. Discussion

The cross-streak plate method showed that the selected potential probiotic strains were found compatible. While assessing potential multi-strain probiotic cultures, it is essential to carry out compatibility tests in order to avoid the combining of strains showing antagonistic effects against each other. The present results are in agreement with those obtained by Sáez et al. [24], who reported that no inhibition halos of selected LAB cell-free supernatants against the other strains were observed, suggesting the absence of antimicrobial substances against each other that could inhibit their development when combined in a mixed culture form. Likewise, Mohamed et al. [30] have revealed that the 5 selected probiotic strains did not show any inhibitory effect to each other are expected to benefit the hosts without interfering with each other under *in vivo* conditions.

Probiotics have been successfully used for the prevention and treatment of various gastrointestinal diseases of humans and animals [31]. The beneficial effect of probiotic strains present in the fermented food products was recognized to have a nutritional and therapeutic effect on human health [22]. Several *in vivo* and *in vitro* studies have demonstrated that probiotics can inhibit *Salmonella*-associated diarrhea [16–18]. In the present study, the relevant functional characteristics of these potential probiotic LAB strains (*Lactococcus lactis* E124*, Lactobacillus plantarum* K132*, Lactobacillus paracasei* K114 and their combinations) showed effective inhibitory activities against *Salmonella* Typhimurium DT104 mainly in coculture experiments (Table 4).

All three potential probiotic strains and their combinations were able to remarkably inhibit the growth of *Salmonella* Typhimurium DT104 under *in vitro* conditions of the coculturing assay. The highest inhibition (100%; *p* < 0.05) was observed with the combination of the three probiotic strains as in the form of mix 4 (*Lactococcus lactis, Lactobacillus plantarum*, and *Lactobacillus paracasei*). In agreement with this study, Adetoye et al. [32] have revealed that *Lactobacillus salivarius* C86 and *Lactobacillus amylovorus* C94 strains obtained from cattle feces were able to inhibit the growth of *Salmonella* spp. completely between 8 and 16 hours of coincubation with no recoverable *Salmonella* spp. in the growth medium. Different authors have also reported a strong inhibition of *Salmonella* spp. by potential probiotic LAB strains in the coculture assay [16, 33, 34]. In line with this, Potočnjak et al. [25] reported that all the tested *Lactobacillus plantarum* strains (A, B, and S1) were able to inhibit (*p* < 0.05) the growth of *Salmonella* Typhimurium at all-time points (6, 12, and 24 h) in the coculture assay. The same author reported that after 24 h of cocultivation, the number of *Salmonella* cells was reduced 1000 times in comparison with *Salmonella* monoculture and the inhibition was most pronounced after 12 h of coincubation and amounted to 97, 98, and 94% by strains A, B, and S1, respectively.

In the present study, oral administration of lactic acid bacteria to model mice caused complete inhibition of the test pathogen, particularly when used in combined form. Oral administration of potential probiotic LAB strains has a beneficial effect on maintaining and improving host health [35]. Viable probiotic strains are used in most probiotic studies, while few studies using heat-killed probiotic bacteria have been reported [36]. Several studies [16, 35, 37] indicated that probiotic LAB strains have protective effects against *Salmonella* infections by involving a number of possible mechanisms. Consequently, the protective mechanisms of inhibition by viable probiotic lactic acid bacteria encompass antimicrobial compounds produced by probiotic bacteria that kill enteric pathogens directly in the gastrointestinal tract [38], enhanced host intestinal immunity by increasing secretory IgA production to eliminate enteric pathogens [39] and competitive inhibition by binding to receptors used by pathogens on epithelial cells such as mannose and glycoproteins [40].

The mortality rate of the challenged mice with *Salmonella* Typhimurium DT104 was prevented by pretreatment with mixed probiotic LAB strains (*Lactococcus lactis, Lactobacillus plantarum* and *Lactobacillus paracasei*) using a mouse model. In agreement with the current study, Moura et al. [41] reported that the survival rate of mice supplemented with *Lactobacillus acidophilus* UFV-H2B20 and then infected with *Salmonella* Typhimurium was significantly (*p* < 0.05) higher in the experimental conventional group (34.6% survival) compared to the control group (0% survival). As observed with other studies [35], to assess the potential anti-*Salmonella* activity, mice were orally supplemented with a mixture of *Lactobacillus plantarum* for 10 days followed by infection with *Salmonella* Typhimurium SL1344. As a result, the survival rate of *Lactobacillus plantarum*-pretreated group was 60% at 15 days after infection, whereas that of the infected group was only 40%. Moreover, Júnior et al. [37] have revealed that the higher survival rate (70%) was observed in mice that were promptly treated with oral administration of *Lactobacillus diolivorans* 1Z and challenged with *Salmonella* Typhimurium in comparison with mice received only water (0% survival) and then challenged with *Salmonella* Typhimurium.

In the present study, the mixed probiotic cultures eliminated the target organism from the feces of probiotic-treated mice groups while administrated at log 6 CFU/ml inoculum level on day 20. However, the elimination of *Salmonella* Typhimurium DT104 took quite a long time in the experimental mice. This could be due to the time required for a probiotic strain to colonize the intestine and play their probiotic roles. On the contrary, mice without probiotics (typ) showed a high population of *Salmonella* Typhimurium DT104 on day 20. In general, regular consumption of fermented products containing probiotic cultures would result in the establishment of these cultures in the human intestine, which will help in rapid elimination of an enteric pathogen in the intestine. Furthermore, the reduction of intestinal *Salmonella* numbers due to the effects of probiotics was reported by different workers [36, 42, 43]. *Salmonella* Typhimurium ATCC 14028 was completely excluded within 23 days in mice when *Lactobacillus rhamnosus* and *Lactobacillus plantarum* were used as probiotics [29]. Similarly, oral administration of a combination of select lactic acid bacteria strains had a significant protective effect on *Salmonella* invasion and inflammation in broiler chicks [28].

Correspondingly, the mixed strains of *Lactobacillus plantarum* had preventive effects against *Salmonella* infection as they decreased *Salmonella*-induced animal deaths in a mouse model [35]. Administering a five-strain probiotic combination as either a milk fermentate or milk suspension for a total of 30 days significantly reduced *Salmonella* Typhimurium infection in probiotic-treated pigs at 15 days after infection [44]. Moreover, the protective effect of *Lactobacillus rhamnosus* GG against *Salmonella* infection in mice was significantly different at 4, 7, and 11 days after inoculation with *Salmonella* Typhimurium C5 [45].

## 6. Conclusion

Ethiopian fermented food products are rich in potential probiotic LAB strains that may exhibit antimicrobial efficacy against foodborne pathogens due to their bactericidal properties. In the present study, the selected potential probiotic LAB strains (*Lactobacillus plantarum* K132*, Lactobacillus paracasei* K114 and *Lactococcus lactis* E124) were able to show a protective effect against *Salmonella* Typhimurium DT104 infection in experimental mice. As a result, oral administration of the selected potential probiotic strains was able to protect mice against infection with *Salmonella* Typhimurium DT104 that suggests their promising potential be used for the production of functional fermented products.

## Figures and Tables

**Table 1:** Source of probiotic strains and *Salmonella* Typhimurium used in this investigation.

Number	Designation	PROB1806	Strains	Source of isolation
1	E 124	E8	*Lactococcus lactis*	*Ergo*
2	K114	G9	*Lactobacillus paracasei*	*Kocho*
3	K132	H8	*Lactobacillus plantarum*	*Kocho*
4	DT104	ATCC	*Salmonella* Typhimurium	EPHI

E = ergo and K = kocho; EPHI: Ethiopian Public Health Institute.

**Table 2:** Preparation of pure and mixed potential probiotic LAB cultures for the coculture assay.

No.	Isolate code	Pure and mixed potential probiotic LAB strains
1	E124	*Lactococcus lactis*
2	K320	*Lactobacillus plantarum*
3	K114	*Lactobacillus paracasei*
4	E124 + K320	*Lac. lactis* + *Lb. plantarum* (mix 1)
5	E124 + K114	*Lac. lactis* + *Lb. paracasei* (mix 2)
6	K320 + K114	*Lb. plantarum* + *Lb. paracasei* (mix 3)
7	E124 + K114	*Lac. lactis* + *Lb. plantarum* + *Lb. paracasei* (mix 4)

E = ergo; K = kocho.

**Table 3:** The interaction between the selected potential probiotic LAB strains.

No	Isolate code	Mixed probiotic strains	Inhibition	Cogrowth
1	E124-K320	*Lac. lactis*-*Lb. plantarum*	−	+
2	E124-K114	*Lac. lactis*-*Lb. paracasei*	−	+
3	K320-K114	*Lb. plantarum*-*Lb. paracasei*	−	+
4	E124-K320-K114	*Lac. lactis*-*Lb. plantarum*-*Lb. paracasei*	−	+

E = ergo; K = kocho. “−” indicates no inhibition halos against each other; “+” indicates able to exist and perform agreeable.

**Table 4:** Coculture assay of probiotic LAB strains against *Salmonella* Typhimurium DT104.

No	Isolate code	Probiotic strains	Inhibition (%)
1	E124	*Lactococcus lactis*	97.11 ± 0.23^b^
2	K320	*Lactobacillus plantarum*	96.50 ± 0.29^c^
3	K114	*Lactobacillus paracasei*	97.27 ± 0.26^b^
4	E124 + K320	*Lac. lactis* + *Lb. plantarum* (mix 1)	99.71 ± 0.02^a^
5	E124 + K114	*Lac. lactis* + *Lb. paracasei* (mix 2)	99.74 ± 0.02^a^
6	K320 + K114	*Lb. plantarum* + *Lb. paracasei* (mix 3)	99.72 ± 0.01^a^
7	E124 + K320 + K114	*Lac. lactis* + *Lb. plantarum* + *Lb. paracasei* (mix 4)	100.00 ± 0.00^a^

E = ergo; K = kocho. Data are means ± SD from three replications and values followed by a different letters within columns indicate significant differences (*p* < 0.05).

**Table 5:** Viable cell counts of *Salmonella* Typhimurium DT104 in fecal material of mice.

Sample	Sampling days	Treatment group		
		Negative control (PBS)	Treated group (pro-typ)	Positive control (typ)
Fecal material of mice	Day 9	0.00 ± 0.00^a^	2.27 ± 0.01^ab^	4.47 ± 0.02^f^
	Day 10	0.00 ± 0.00^a^	2.30 ± 0.03^a^	4.52 ± 0.04^e^
	Day 11	0.00 ± 0.00^a^	1.93 ± 0.04^abc^	4.56 ± 0.01^bcd^
	Day 12	0.00 ± 0.00^a^	1.90 ± 0.07^abc^	4.55 ± 0.02^cde^
	Day 13	0.00 ± 0.00^a^	1.88 ± 0.04^abc^	4.58 ± 0.02^abc^
	Day 14	0.00 ± 0.00^a^	1.82 ± 0.05^bcd^	4.61 ± 0.00^a^
	Day 15	0.00 ± 0.00^a^	1.74 ± 0.06^cd^	4.60 ± 0.01^a^
	Day 16	0.00 ± 0.00^a^	1.65 ± 0.07^cd^	4.59 ± 0.00^ab^
	Day 17	0.00 ± 0.00^a^	1.65 ± 0.07^cd^	4.56 ± 0.01^bcd^
	Day 18	0.00 ± 0.00^a^	1.39 ± 0.13^d^	4.56 ± 0.01^bcd^
	Day 19	0.00 ± 0.00^a^	0.50 ± 0.71^e^	4.54 ± 0.01^de^
	Day 20	0.00 ± 0.00^a^	0.00 ± 0.00^f^	4.53 ± 0.01^de^
	Day 21	0.00 ± 0.00^a^	0.00 ± 0.00^f^	4.52 ± 0.02^e^

PBS = PBS = phosphate-buffered saline;pro + typ = both mixed probiotic cultures and *Salmonella*; typ = mono *Salmonella* Typhimurium DT104. Data are means ± SD from three replications and values followed by different letters within columns indicate significant differences (*p* < 0.05).

## Data Availability

The data used to support the findings of this study are included in the article and are available (the SAS data) from the corresponding author upon request.
